# Concentrations and Sources of Airborne Particles in a Neonatal Intensive Care Unit

**DOI:** 10.1371/journal.pone.0154991

**Published:** 2016-05-13

**Authors:** Dusan Licina, Seema Bhangar, Brandon Brooks, Robyn Baker, Brian Firek, Xiaochen Tang, Michael J. Morowitz, Jillian F. Banfield, William W. Nazaroff

**Affiliations:** 1 Department of Civil and Environmental Engineering, University of California, Berkeley, California, United States of America; 2 Department of Earth and Planetary Sciences, University of California, Berkeley, California, United States of America; 3 Division of Newborn Medicine, Magee-Womens Hospital of UPMC, Pittsburgh, Pennsylvania, United States of America; 4 Department of Surgery, University of Pittsburgh School of Medicine, Pittsburgh, Pennsylvania, United States of America; 5 Division of Pediatric Surgery, Children's Hospital of Pittsburgh, Pittsburgh, Pennsylvania, United States of America; Columbia University, UNITED STATES

## Abstract

Premature infants in neonatal intensive care units (NICUs) have underdeveloped immune systems, making them susceptible to adverse health consequences from air pollutant exposure. Little is known about the sources of indoor airborne particles that contribute to the exposure of premature infants in the NICU environment. In this study, we monitored the spatial and temporal variations of airborne particulate matter concentrations along with other indoor environmental parameters and human occupancy. The experiments were conducted over one year in a private-style NICU. The NICU was served by a central heating, ventilation and air-conditioning (HVAC) system equipped with an economizer and a high-efficiency particle filtration system. The following parameters were measured continuously during weekdays with 1-min resolution: particles larger than 0.3 μm resolved into 6 size groups, CO_2_ level, dry-bulb temperature and relative humidity, and presence or absence of occupants. Altogether, over sixteen periods of a few weeks each, measurements were conducted in rooms occupied with premature infants. In parallel, a second monitoring station was operated in a nearby hallway or at the local nurses’ station. The monitoring data suggest a strong link between indoor particle concentrations and human occupancy. Detected particle peaks from occupancy were clearly discernible among larger particles and imperceptible for submicron (0.3–1 μm) particles. The mean indoor particle mass concentrations averaged across the size range 0.3–10 μm during occupied periods was 1.9 μg/m^3^, approximately 2.5 times the concentration during unoccupied periods (0.8 μg/m^3^). Contributions of within-room emissions to total PM_10_ mass in the baby rooms averaged 37–81%. Near-room indoor emissions and outdoor sources contributed 18–59% and 1–5%, respectively. Airborne particle levels in the size range 1–10 μm showed strong dependence on human activities, indicating the importance of indoor-generated particles for infant’s exposure to airborne particulate matter in the NICU.

## Introduction

Hospital-acquired infections are recognized as a major source of morbidity and mortality among hospital patients of all ages [1]. According to the CDC [2], 1 in 25 patients acquire at least one infection while hospitalized. It is challenging to know the sources of infection and mechanisms of transmission of hospital-acquired infections. Several studies have identified diverse sources and reservoirs of causative organisms, including room air [3,4], indoor surfaces [5–8], and room occupants (i.e., personnel and visitors [9] and patients themselves who can cause autogenous infection [10]). A recent review by Beggs et al. [11] highlights the importance of environmental factors.

Premature infants in the neonatal intensive care unit (NICU) have underdeveloped immune systems that could make them susceptible to deleterious health consequences from adverse environmental exposures. There are growing needs for NICUs owing to increasing premature birth rates. There is also clear evidence indicating that NICU environments may contain reservoirs of clinically important pathogens. For example, a recent review [12] found that as much as 63% of extremely preterm infants develop life-threatening infections, possibly owing to environmental exposures to potential pathogens.

Although inhaling indoor air is known to be a generally important exposure route associated with adverse health effects, there is as yet limited knowledge available on concentrations and sources of airborne particles in NICU environments. Also, hospital hygiene protocols may undervalue the potential importance of the airborne transmission route, which could result in inappropriate or insufficient intervention to mitigate indoor microbial burdens and their transmission to neonates. A growing need is evident for more comprehensive investigations of NICU environments to develop better knowledge of the sources of airborne particles and the potential for exposure to them by preterm infants.

Only a few prior studies have investigated air quality parameters in a NICU environment. These focused on cleaning/renovation activities and the mechanical ventilation system as potential sources. Bokulich et al. [13] highlighted the importance of routine cleaning for controlling microbes in the NICU and for minimizing exposures of susceptible infants to potential pathogens. Shimono et al. [14] found that vigorous cleaning and adequate provision of ventilation are required for outbreak control in NICUs. Renovation works in a NICU have been shown to significantly increase concentrations of *Aspergillus spp*. [15]. It has been recognized that infectious disease spread can occur through heating ventilation and air conditioning (HVAC) systems because of colonization with nosocomial pathogens [16]. Implementing efficient air filtration and/or ultraviolet germicidal irradiation (UVGI) in an HVAC system decreases microbial burden in the NICU [17,18]. Environmental factors such as air temperature, relative and absolute humidity, as well as architectural design, have been shown to affect growth and survival responses of bacteria, viruses and fungi in various types of health-care facilities and under different operating conditions [19–23].

To our knowledge, only one prior study [9] reported preliminary evidence in support of the hypothesis that human activities are significant contributors of particles larger than 2 μm in the NICU setting. That study compared two contrasting NICU designs, open-style vs. private family units, in terms of air quality performance and patient medical progress. The results showed that private units had lower levels of noise, airborne particles and carbon dioxide, as well as exhibiting enhanced infant medical progress. These features were attributed to reduced activities associated with health-care delivery. The study answered a few important questions on air quality in a NICU environment, but left open many other questions. For instance, the study did not differentiate among different particle sizes, which is certainly a relevant factor for environmental fate and for health-pertinent exposure assessments.

The current study constitutes the major part of our larger effort to improve knowledge about the sources, dynamic behavior, and fate of airborne particles in the NICU environment. Our pilot investigation [24] utilized short-term observational monitoring and researcher-manipulated conditions to make important inferences about sources of airborne particles in a NICU baby room and about conditions in an unoccupied incubator. A valuable finding in that effort is that concentrations of large particles increased with the number of occupants and with the duration and vigor of activities. Also, within-incubator particle concentration levels reached up to 60% of those in the room at times when the incubator humidification was inactive. Steam humidification was identified as a significant source of airborne particles smaller than 5 μm within the infant incubator.

The present study builds upon the lessons learnt from the pilot study. It is the first to report a baseline characterization of airborne particle concentrations, sources and dynamics in a normally functioning NICU monitored throughout a one-year period. The primary study objectives are: (i) to characterize particle number concentrations and to determine spatial and temporal differences of indoor environmental parameters across an ordinarily operating NICU; and (ii) to identify and quantify sources of size-resolved airborne particles in private-style NICU rooms. The results of this study could find potential application in exposure and health-risk assessments for infants in NICU environments. It could also prove valuable for the identification and characterization of interventions to reduce indoor airborne particle concentrations and their transfer to premature infants.

## Materials and Methods

### Study site

The field-monitoring portion of the study was conducted at the 6-story Magee-Womens Hospital of the University of Pittsburgh Medical Center in the United States. Measurements were made in a private-style NICU located at the first level of the hospital and configured to provide a personalized care-giving environment and services for each infant and family member. The entire floor area of the NICU (1775 m^2^), one of the largest in the USA, is covered with hard tiles which were cleaned approximately every 24 hours by using microfiber mops that were replaced after each use. All individual baby rooms were cleaned daily and there were no evident signs of dust accumulation. All the furnishings and materials used in the NICU were free of substances known to be teratogenic, mutagenic, carcinogenic or otherwise harmful to human health, as recommended for NICU design [25]. Smoking and pets were not allowed in the unit. Hand-washing was required for staff and visitors upon entering and exiting the baby room; however, exchange of street clothing and shoes was not mandatory. Scrubs worn by the hospital staff were their own personal property and were typically worn into and out of the hospital without changing. Visitors were not required to wear any cover for their hair or mouth and nose.

**Fig 1:**
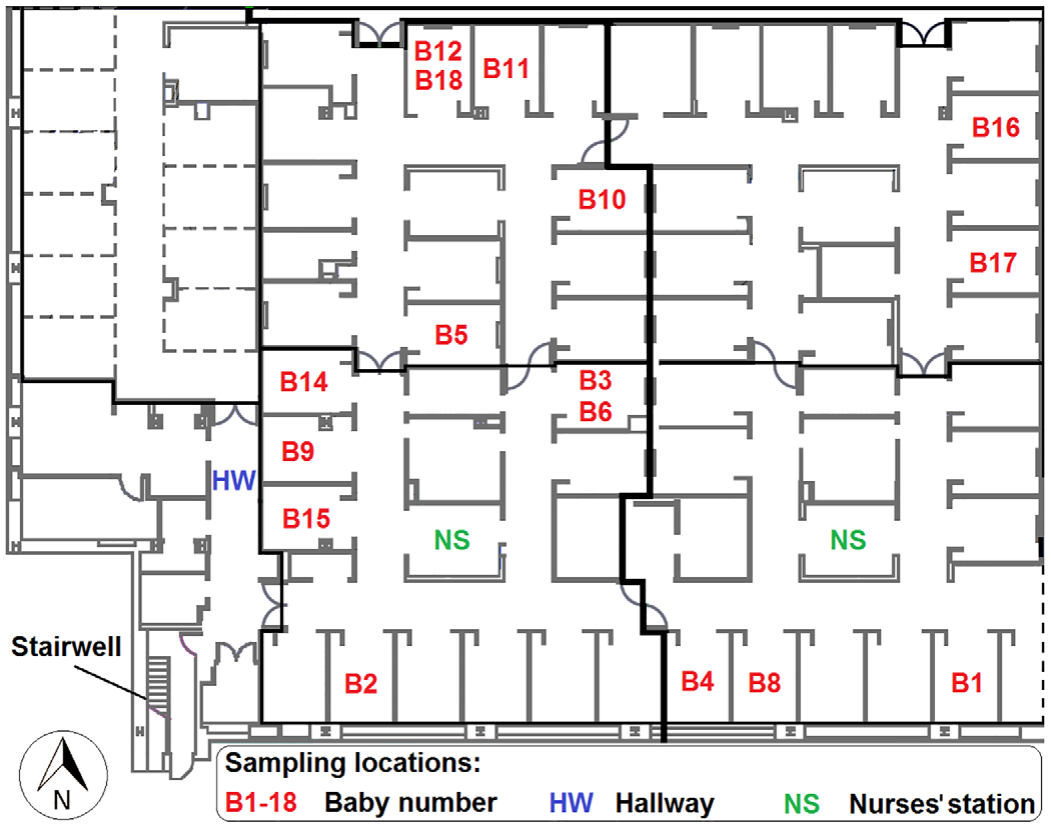
Schematic layout of the NICU, located on building level 1 and served by its own air-handling unit. Most of the NICU patient rooms were similar in size (about 33 m^3^). Sampling sites were in baby rooms (red), hallway (blue) and nurses’ stations (green).

The physical layout of the NICU consisted of multiple private-style baby rooms, connecting hallways, and two nurses’ stations, as schematically shown in Fig 1. Physical dimensions of the baby room were 4.6 × 2.9 × 2.4 m (L × W × H), and each was designed to allow unobstructed passage of equipment and personnel to the hallway. The only physical boundary between the baby room and the hallway was a curtain that was occasionally drawn but was usually open. Each baby room was equipped with an incubator, chairs for visitors, sink, trash can, drawers and shelves for hospital equipment. The nurse’s station was relatively densely occupied by caregivers and visitors. It was equipped with furniture and electronic devices such as computers and printers. The NICU had no operable windows or any visible signs of air infiltration; however, the hallway at the southwest façade had an exit doorway connecting to a stairwell that led out of the building (Fig 1).

### Experimental design and analysis

We conducted a yearlong air quality study in a normally functioning NICU, with the main focus on airborne particles. Sampling was performed with 1-min time resolution to capture the dynamic processes and responses to rapidly changing conditions. Airborne particles larger than 0.3 μm were quantified in six size categories based on light scattering intensity. Distinguishing among individual particle-size groups enabled us to apportion each group to corresponding particle source categories. The contributions of human activities to within-room particle generation were quantitatively compared with the estimated levels of particles entering the room from the hallway and also with particles entering from outdoors via the mechanical ventilation system.

Two or three sets of instruments were concurrently deployed to obtain information about airborne particle concentrations, CO_2_ levels, dry-bulb temperature, relative humidity, and presence or absence of occupants. One set of instruments was located in a baby room that housed one premature infant at a time. In all, 16 infant-room periods were monitored, denoted with B1-B18 (Fig 1). The only prerequisite was that selected babies had a gestational age of 32 weeks or less and a birth weight less than 1250 g. Such infants would typically spend the first month or more of life in an incubator in the NICU. Concurrently with sampling in a baby room, a second monitoring station was operated nearby, either in the hallway or at the nurses’ station. This second station provided important information from which to glean the relative contributions of local indoor versus general indoor versus outdoor concentrations to particle levels in the baby room. In a few cases, two baby rooms were simultaneously sampled when a third set of instruments was employed.

The measurement campaign spanned one year (14 October 2013–28 October 2014), allowing us to explore seasonal variation. The total number of sampling days was 182. A nominal measurement time per baby was 3 weeks, which corresponded to 12 sampling days with 4 weekdays of continuous sampling per week. For each monitored week, measurements were initiated on Monday at 12:00 and continued until Friday 12:00 (Eastern time zone). The number of sampling days per baby was subject to change owing to factors such as instrument malfunction, acquired baby illness, availability of rooms and public holidays. The timing and duration of measurements for each baby are summarized in Table 1.

**Table 1:** Experimental schedule and year-long descriptive data of human occupancy load, dry-bulb temperature, relative humidity and carbon dioxide levels measured in baby rooms (BR) and hallway (Hall). ^1^,^2^

Baby ID	Startdate	Days	Occupancy (mean, %)		T (mean ± S.D., °C)		RH (mean ± S.D., %)		CO_2_ (mean ± S.D., ppm)	
			BR	Hall	BR	Hall	BR	Hall	BR	Hall
B1	14/10/13	12	16		24.1 ± 0.7		40 ± 2		466 ± 31	
B2	28/10/13	12	31		24.5 ± 0.7		40 ± 2		452 ± 38	
B3	3/12/13	10	22	14	23.9 ± 0.2	24.2 ± 0.2	39 ± 1	34 ± 3	463 ± 22	455 ± 18
B4	9/1/14	12	22	13	25.6 ± 0.6	24.5 ± 1.0	36 ± 2	34 ± 4	490 ± 30	434 ± 19
B5	27/1/14	14	32	13	23.9 ± 0.4	24.1 ± 1.3	40 ± 2	35 ± 3	467 ± 33	440 ± 25
B6	3/3/14	11	16	13	24.1 ± 0.6	23.8 ± 0.4	39 ± 2	38 ± 3	471 ± 23	433 ± 23
B8	7/4/14	12	27	12	24.6 ± 0.6	23.7 ± 0.4	38 ± 2	40 ± 2	467 ± 36	419 ± 44
B9	28/4/14	12	19	11	23.7 ± 0.3	23.2 ± 0.3	41 ± 2	42 ± 2	439 ± 48	439 ± 43
B10	9/6/14	12	33	13	23.3 ± 0.2	23.9 ± 0.1	45 ± 1	43 ± 1	513 ± 60	485 ± 50
B11	12/6/14	12	24	13	24.5 ± 0.5	23.8 ± 0.3	43 ± 2	44 ± 3	542 ± 59	485 ± 46
B12	8/7/14	14	28	13	23.5 ± 0.8	24.1 ± 0.5	43 ± 2	42 ± 2	501 ± 61	446 ± 54
B14	31/7/14	13	20	12	22.9 ± 0.7	23.2 ± 0.6	45 ± 2	44 ± 2	456 ± 53	458 ± 49
B15	14/8/14	5	40	11	23.7 ± 0.2	23.1 ± 0.2	44 ± 1	45 ± 1	512 ± 60	463 ± 46
B16	25/8/14	11	16	14	23.3 ± 0.6	24.5 ± 0.3	44 ± 2	42 ± 1	468 ± 54	456 ± 52
B17	23/9/14	11	18	13	23.9 ± 0.4	24.2 ± 0.4	41 ± 2	41 ± 1	481 ± 40	426 ± 38
B18	13/10/14	9	22	13	23.6 ± 0.6	24.0 ± 0.2	41 ± 2	41 ± 2	443 ± 42	417 ± 28
	**Mean**		**24**	**13**	**23.9 ± 0.5**	**23.9 ± 0.4**	**41 ± 2**	**40 ± 2**	**477 ± 43**	**450 ± 38**

^1^Sampling in the baby rooms B1, B2 and the first three days of B3 was conducted along with measurements at the nurses’ station. The results of sampling at the nurses’ station were analyzed and reported independently (S1 File, S2 Fig) and excluded from the present table and from further analyses. Babies 7 and 13 had to be transferred to another hospital owing to acquisition of NEC (necrotizing enterocolitis); they were excluded from this study. Baby 15 acquired NEC but was retained for the analysis with reduced number of sampling days. Unexpected events (HVAC filter maintenance) were also excluded from the current analysis and are reported separately (S1 File, S3 Fig). The size-resolved particle number concentrations without applying data post-processing (including data from nurses’ station and HVAC filter maintenance) are reported elsewhere (S2 Table).

^2^The results of occupancy, dry-bulb temperature, relative humidity and carbon dioxide are 1-min mean and standard deviation acquired during day and night sampling. Occupancy data for B1 and B2 were obtained based on a light sensor with detectable range of 12 m; the remaining data were recorded with the occupancy sensor described in Materials and Methods.

### Mechanical ventilation

The NICU is served by a central HVAC system equipped with an economizer and a high-efficiency particle filtration system. A single dedicated air handing unit (AHU) served the entire NICU with an outdoor air intake situated at the southwest corner of the hospital. The AHU delivered 42,500 m^3^/h to the NICU as a mixture of recirculated and outdoor air. That volumetric flow rate corresponds to a turnover of about 10 interior volumes per hour with the design minimum being one-third outdoor air; the total flow is deemed sufficient to maintain well-mixed conditions in each room. The economizer provides “free-cooling” that supplies up to 90% outdoor air, based on the outdoor air dry-bulb temperature (90% of outdoor air is supplied when the outdoor air dry-bulb temperature is ~10°C). After mixing recirculated air with filtered outdoor air, the air is cooled with coils that are treated with ultraviolet germicidal irradiation (UVGI) to kill or inactivate microorganisms. The air mixture is subsequently heated and humidified in the AHU and reheated at the terminal box according to local thermostat demand. The secondary “trim” humidifiers accurately controlled the final zone humidification to maintain the target value of ~ 40% RH. The water for humidification was treated steam from boilers. The outdoor air was first treated with MERV 8 pleated filters, which were replaced based on limiting values for the measured pressure drop across filter sections. The final stage of filtration applied to both outdoor and recirculated air was achieved through high-efficiency particulate arrestance (HEPA) filters. The conditioned air was introduced to the NICU rooms through ceiling-mounted air diffusers that were situated to minimize draft risk near the baby incubators.

### Experimental equipment

The following parameters were measured continuously during weekdays with 1-min resolution: indoor airborne particle concentrations, carbon dioxide (CO_2_) levels, dry-bulb temperature, relative humidity, and presence or absence of occupants. All samplers (except occupancy sensors) were placed in an air-cooled, foam-coated enclosure situated on the floor to provide security and to limit instrument noise. The sampling inlet extended vertically above the enclosure to 0.8 m height through an electrically conductive tube. Although lower than the incubator height, this sampling height is expected to reflect room-averaged conditions well. For example, prior studies show a vertical concentration gradient due to particle resuspension from the floor to be negligible for particles of 10 μm and less [26].

Real-time, size-resolved optical particle counters (Model Met One HHPC 6+, Beckman Coulter Life Sciences, Palatine, IL, USA) were employed to measure concentrations of airborne particles in the NICU. These portable (27 × 10 × 5 cm) particle counters have a detectable minimum particle diameter of 0.3 μm. Number concentrations are reported in six size bins based on optical diameter (0.3–0.5; 0.5–1; 1–2; 2–5; 5–10; and >10 μm).

Real-time CO_2_ measurements were made by means of gas analyzers (Model SBA-5, PP Systems, Amesbury, MA, USA; and model 820, LI-COR Biosciences, Lincoln, NE, USA). The purpose of acquiring CO_2_ data was to gain insight about the human-associated particle generation through acquisition of data reflecting the level of human metabolic emissions, which would in turn be associated with the number of occupants, duration of occupancy, and intensity of activities performed.

Because supply air through the ventilation system is well filtered and because there are no other known strong indoor sources (such as unvented combustion), we anticipated that human occupants and their movement would be important contributors to airborne particle levels. Consequently, we monitored occupancy through the use of infrared motion sensors (Model HOBO UX90-005). The occupancy sensors were installed near the open doorway of the monitored room (facing inward into the room) and also in the hallway location to monitor the presence or absence of people within the detection range of 5 m. Temperature and relative humidity data also were sampled and recorded using HOBO U12 data loggers (Onset Computer Corp., Bourne, MA, USA).

### Quality assurance

To accurately compare results obtained with four optical particle counters, the data were corrected using adjustment factors obtained from side-by-side tests of instrument performance (S1 Table). Flow rate checks were conducted every 2–3 months and either (a) all flows were within 5% of the manufacturer-specified setpoint (2.83 L/min), or (b) instruments with a low flow were repaired before redeploying. Adjustment of CO_2_ data was not necessary, since the instruments had an error less than 1% for the calibrated range of values. Relative differences in CO_2_ levels up to 15 ppm were considered to fall within measurement uncertainty.

### Data analysis: Particle number and mass concentration estimates

Total particle number (TPN) concentrations were obtained by summing number concentrations of particles from the six size bins. The PM_10_ mass levels were estimated as the sum of the computed particle mass concentrations in size bins larger than 0.3 μm and smaller than 10 μm. To perform number to mass conversion, these assumptions were made: (i) particles are spherical and their density is constant at 1000 kg/m^3^; and (ii) mass-weighted size distribution, d*M*/d(log *d*_p_), is constant within each particle size bin. As the average density of particles is likely to be in the range 1000 to 2500 kg/m^3^, the PM_10_ mass concentrations reported here should be viewed as lower-bound estimates [27]. Particles with optical diameter less than 0.3 μm were below the detection limit of the particle counters. However, their “invisibility” was deemed to contribute negligibly to the total mass concentration of airborne particles owing to the absence of evident submicron particle sources indoors and the effective filtration of particles of outdoor origin. Concentrations, emission rates, and dynamic behaviour of particles below 0.3 μm should be studied in the future, as excessive presence of ultrafine particles does pose potential health risks [28].

## Results and Discussion

### Summary of descriptive data

The measurements performed in baby rooms and in a nearby hallway provided more than 5 million data points acquired during the 1-year field-campaign period (S1 Fig).

The time-averaged PM_10_ mass concentrations in the individual baby rooms were generally low, not exceeding 5 μg/m^3^ (mean ± standard deviation = 2.9 ± 0.8 μg/m^3^), which we attribute to efficient particle filtration, effective building hygiene protocols and relatively low occupancy. The indoor PM_10_ levels in the NICU were substantially below typical concentrations measured in other indoor environments, such as residences (median = 34.7 μg/m^3^) and especially schools (median = 102 μg/m^3^), as reported in studies reviewed by Morawska et al. [29].

Table 1 summarizes environmental parameters such as dry-bulb temperature, relative humidity and CO_2_ levels across all baby rooms and hallway sampling periods. The detected occupancy load in baby rooms (excluding the baby) ranged from 16 to 40% and always exceeded the hallway occupancy, which fluctuated within a range of 11–14%. The dry bulb temperature and relative humidity were controlled within a relatively narrow band by the HVAC system. The CO_2_ levels were within the range typical of well-ventilated indoor environments [30] and showed moderate variability. Factors influencing the variability in CO_2_ levels include variable air recirculation rates (more recirculation and less outside air in the summer) and uneven CO_2_ generation by human metabolism within the NICU. The CO_2_ levels exhibited a weak association with the local occupancy load.

### Spatial variation and statistical distribution

**Fig 2:**
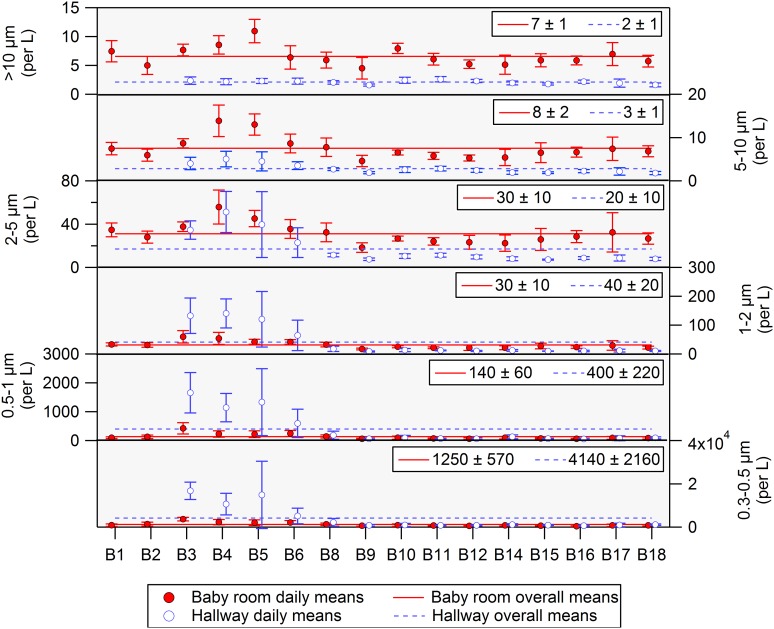
Size-resolved particle number concentrations measured in baby rooms and in the hallway. Data are averages of 1-day mean concentrations (± standard deviation). Boxed values in each frame indicate overall means obtained during the 1-year monitoring campaign. Note that due to sampling at 0.8 m height, reported number concentration for particles >10 μm may be overestimated compared to room-average values [26].

Fig 2 presents mean, size-specific particle concentrations measured in all baby rooms and the hallway. Considering particles larger than 2 μm, average concentrations were higher in baby rooms than in the hallway throughout the year, a result that is aligned with occupancy as a prominent source and the intensity of occupancy being higher in the baby rooms than in the hallway. Seasonal fluctuations in particle number concentrations and CO_2_ levels were relatively small, excluding data from baby rooms B3, B4 and B5. A slight decrease in particle number concentrations during the summer period (corresponding to babies B11-B14) is attributed to an increased proportion of supply air being recirculated within the ventilation system (Table 1; S2 Table).

Within three baby rooms (B3, B4 and B5), particle levels were notably higher compared to other baby rooms. We speculate that the cause was concurrent wintertime construction being undertaken near the southwest façade of the hospital (only during B3, B4 and B5), close to the outdoor air intake. It may be that during the construction period, elevated levels of outdoor particles were introduced to the NICU through the HVAC system and via particle infiltration. As seen in Fig 2, the seasonal pattern of elevated particle number concentrations (babies B3-B6) was more discernible in the hallway and within the smaller particle-size fractions. We suspect that the explanation for higher levels in the hallway during these times relates to the building’s physical configuration and ventilation system design. The stairwells are not mechanically ventilated. Consequently, air in the stairwell infiltrates from outdoors solely via the exit doorway (Fig 1), yielding an air-exchange rate that is likely to be low. Outdoor particle infiltration and persistence (the infiltration factor) is a function of a size-specific particle penetration efficiency and deposition rates on indoor surfaces. In a low air-exchange environment, particles at the smaller end of the size range that we monitored would have a higher infiltration factor [31]. The small particles that remain airborne could penetrate into the hallway from the stairwell, possibly through air leakage from the frequently opened door that connects the stairwell to the interior of the hospital or through air leakage around the closed door.

**Fig 3:**
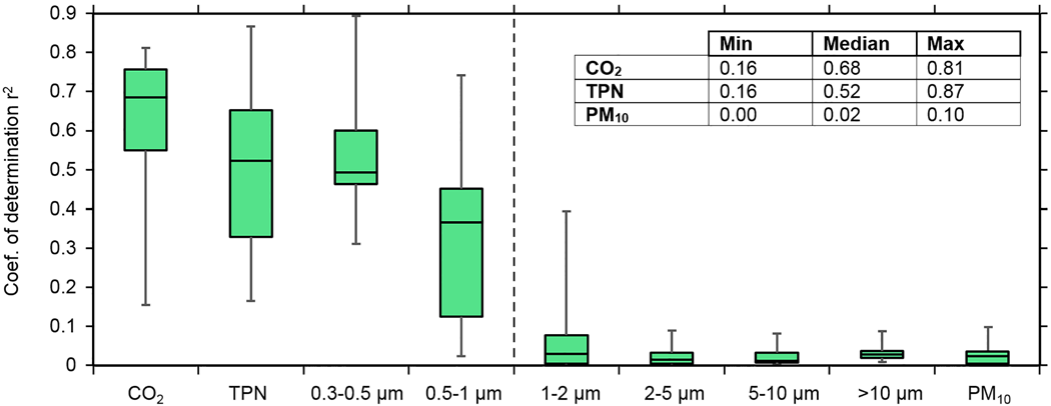
Coefficient of determination between baby room and hallway CO_2_ levels, total particle number (TPN), size-specific PN concentrations and PM_10_ mass. The results are obtained based on 5-min mean concentrations. Box plots indicate minimum, 1^st^ quartile, median, 3^rd^ quartile and maximum values considering data from the whole monitoring campaign.

Spatial parameter correlations between the monitored baby room and the hallway were strong for CO_2_ levels, total particle number (TPN) concentrations, and size-resolved particle concentrations in the submicron size ranges, as shown in Fig 3. Submicron particles contribute substantially to TPN, but do not strongly influence particle mass concentrations. Consequently, the submicron particles exhibit similar correlation factors as TPN, especially in the smallest monitored particle size-range (0.3–0.5 μm). Conversely, correlations were weak for supermicron particle levels and especially for PM_10_ mass (Fig 3), which is mainly driven by larger particles. Evidence from the spatial correlation analysis supports an interpretation that indoor TPN levels were controlled by penetration from outdoor air (and therefore highly correlated between the two indoor monitoring sites). Conversely, the PM_10_ levels, which correlated poorly between the two indoor sites, were primarily derived from occupancy.

There was not a strong link between CO_2_ levels and local occupancy in individual baby rooms (Table 1). That result reinforces and helps to interpret the finding of high CO_2_ spatial correlations between the paired indoor monitoring locations. Strong spatial correlations exist because of several factors: 1) free transport of CO_2_ between NICU rooms due the absence of doors; 2) balanced propagation of CO_2_ throughout the NICU via recirculating airflow in the HVAC system; and 3) the transport without loss of CO_2_ through all ducted airstreams (in contrast to the effective particle filtration from recirculating flow). Therefore, CO_2_ emissions anywhere in the NICU would elevate indoor levels throughout the NICU, whereas indoor particle emissions, particularly in the supermicron particle-size fractions, would tend to have a more localized influence on particle concentrations.

The high spatial correlation for submicron particles was even more pronounced between two baby rooms when they were simultaneously sampled (S4 Fig). Based on 9 overlapping sampling days, simultaneous monitoring in baby rooms in B10 and B11 yielded coefficients of determination (*r*^2^) ranging from 0.1 (for particles larger than 10 μm) to 0.94 (for the 0.3–0.5 μm particle-size fraction). The CO_2_ level in both rooms exhibited a high degree of correlation (*r*^2^ = 0.8), indicating the dominant influence of supply air.

### Temporal variation of CO_2_, TPN, PM_10_ mass and human occupancy

**Fig 4:**
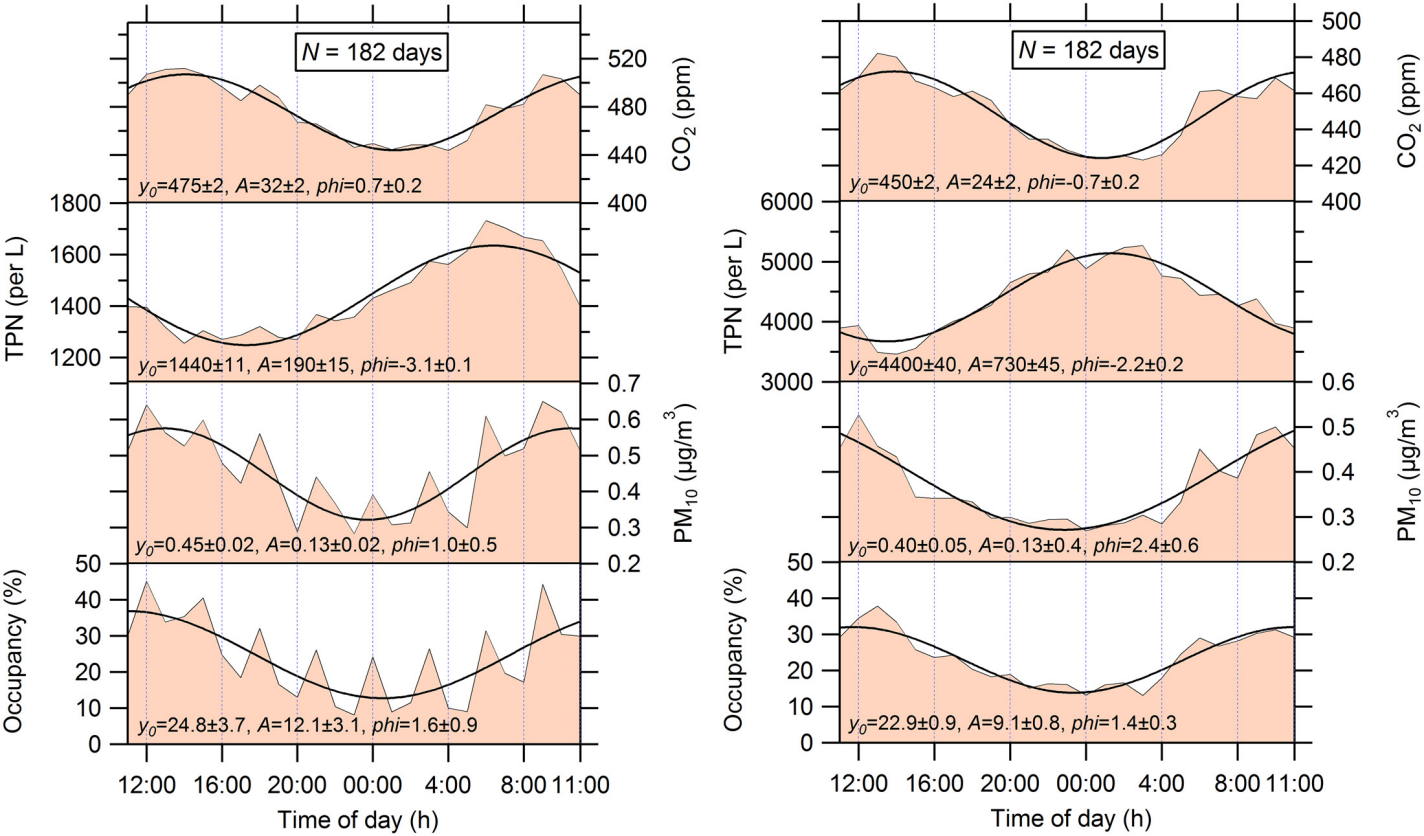
Daily 1-h mean concentrations of CO_2_, total particle number (TPN), PM_10_ mass and human occupancy over the full monitoring period measured in all baby rooms (left) and in the hallway (right). A least-squares best-fit sinusoidal curve (Eq 1) is shown along with function coefficients and standard deviations.

Fig 4 illustrates the effect of within-room diurnal occupancy patterns on hourly mean PM_10_, TPN and CO_2_ levels. The plots were produced by creating hourly mean data, which were subsequently averaged across all baby rooms and across the full study period. All parameters exhibited a diurnal cyclic variation, which was statistically described using a sinusoid (Eq 1):

$$y=y_{0}+A\text{sin}\left(2\pi x+\varphi \right)$$ (1)

The response variable, *y*, is the time-of-the-day averaged value of occupancy, PM_10_, TPN or CO_2_ level. The mean of *y* is *y*_0_, *A* is a maximum span from the mean, *x* is time of day, and *φ* is the phase shift. The least squares best-fit sinusoidal curves are plotted in Fig 4 along with the time-averaged values. The CO_2_ levels followed expected diurnal occupancy patterns: lower overnight and elevated during the day. The daily CO_2_ level rise is mainly a consequence of metabolic CO_2_ generation by occupants anywhere in the NICU. Fig 4 exhibits this pattern, as CO_2_ concentrations in the baby rooms and in the hallway were similar and varied in a comparable manner. By contrast, the influence of occupancy was particularly evident for PM_10_ mass in the baby rooms, clearly indicating a tight correlation between human presence and emissions of coarse particles, most likely from a combination of shedding (from skin, hair and clothing) and resuspension (from the floor and other contact surfaces) [32]. The occupancy data exhibit a “shark-tooth” pattern, peaking every 3 hours, especially during night, with fewer visitors. The intermittent peaks in occupancy correspond to the regular feeding time of babies by hospital personnel. The correlation between occupancy and PM_10_ mass in the hallway was also high, despite the absence of a synchronous activity pattern as in the baby rooms. On the other hand, the diurnal TPN trend, dominated by fine particles, was minimally affected by occupancy. The TPN concentrations increased during the night, when the economizer supplied more outdoor air because of the lower outdoor dry-bulb temperature. This evidence points toward a possible interpretation: the levels outdoors were the predominant influence on indoor TPN levels. This observation corresponds to expectations, given the reduced deposition loss coefficient and filtration efficiency for particles in the size range 0.3–1 μm [33], which can lead to a greater fractional penetration of particles of outdoor origin.

Fig 4 exhibits noteworthy differences in TPN levels and a peak phase-shift between baby rooms and the hallway. These indicators may signal two distinct pathways of particle intrusion indoors. Transport through the HVAC system likely dominates as a TPN source in the baby rooms. However, it appears that an important mechanism of particle intrusion into the hallway was through infiltration from the nearby stairwell, particularly during the construction period. The large discrepancies in TPN between baby rooms and hallway suggest the possibility to reduce indoor levels of small particles by means of ensuring a tight building envelope coupled with effective pressure management to minimize infiltration.

### Relative source contributions to within-room particle concentrations

**Fig 5:**
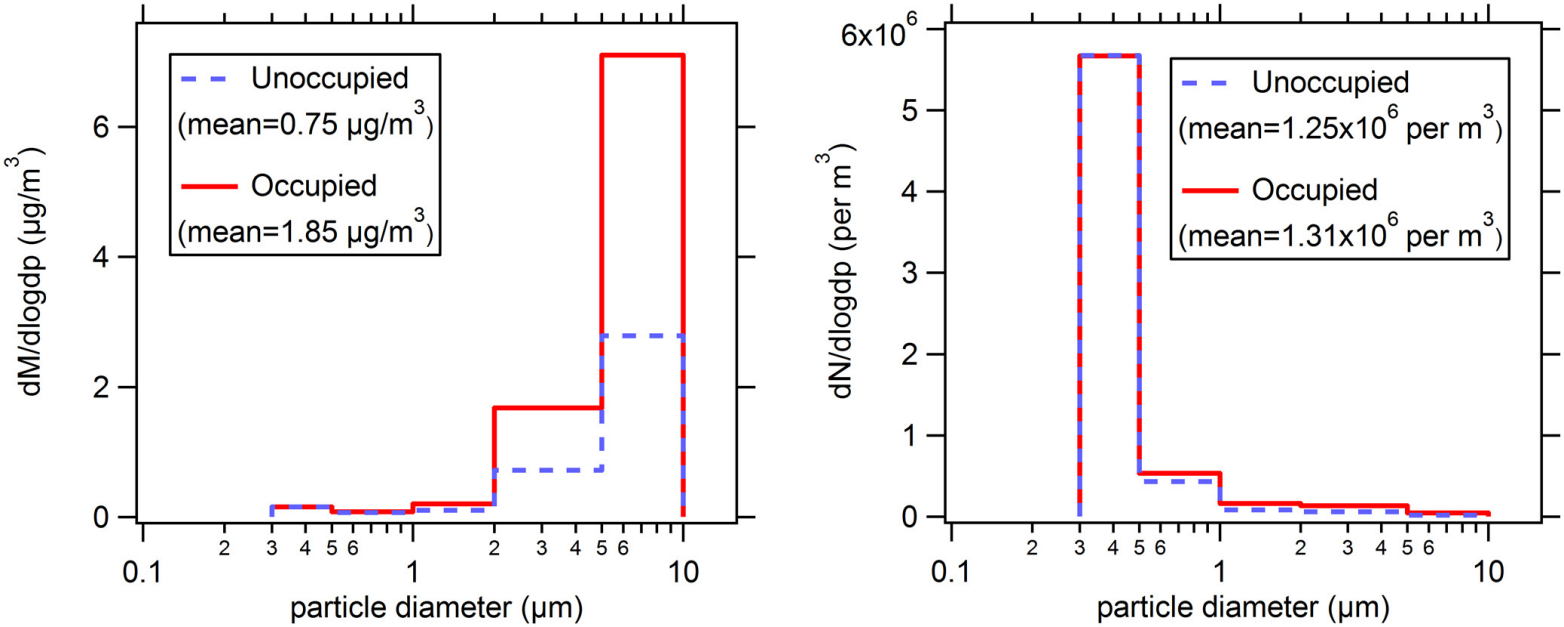
Size distributions of total particle number (left) and mass (right) concentrations averaged across all baby rooms separately considering occupied and unoccupied periods. The data from the largest size channel of the optical particle counter was omitted and an upper limit of 10 μm diameter was adopted.

Fig 5 shows normalized size distributions of indoor particle number and mass concentrations recorded in all baby rooms during occupied and unoccupied conditions. Human presence did not materially influence total particle number concentration but did result in a distinct increase in airborne particle mass concentration. This strong association was discernible within size fractions larger than 1 μm. The recorded difference between submicron particle number concentrations during occupied and vacant periods was less than 5%, indicating that supply from outdoor air played a predominant role for this particle metric. By contrast, mean indoor particle mass concentrations averaged across the size range 0.3–10 μm was 1.85 μg/m^3^ during the occupied periods, approximately 2.5 times as high as levels during unoccupied conditions (0.75 μg/m^3^). These data reveal important features about the contribution of room occupancy to the supermicron mass concentrations of airborne particles.

**Fig 6:**
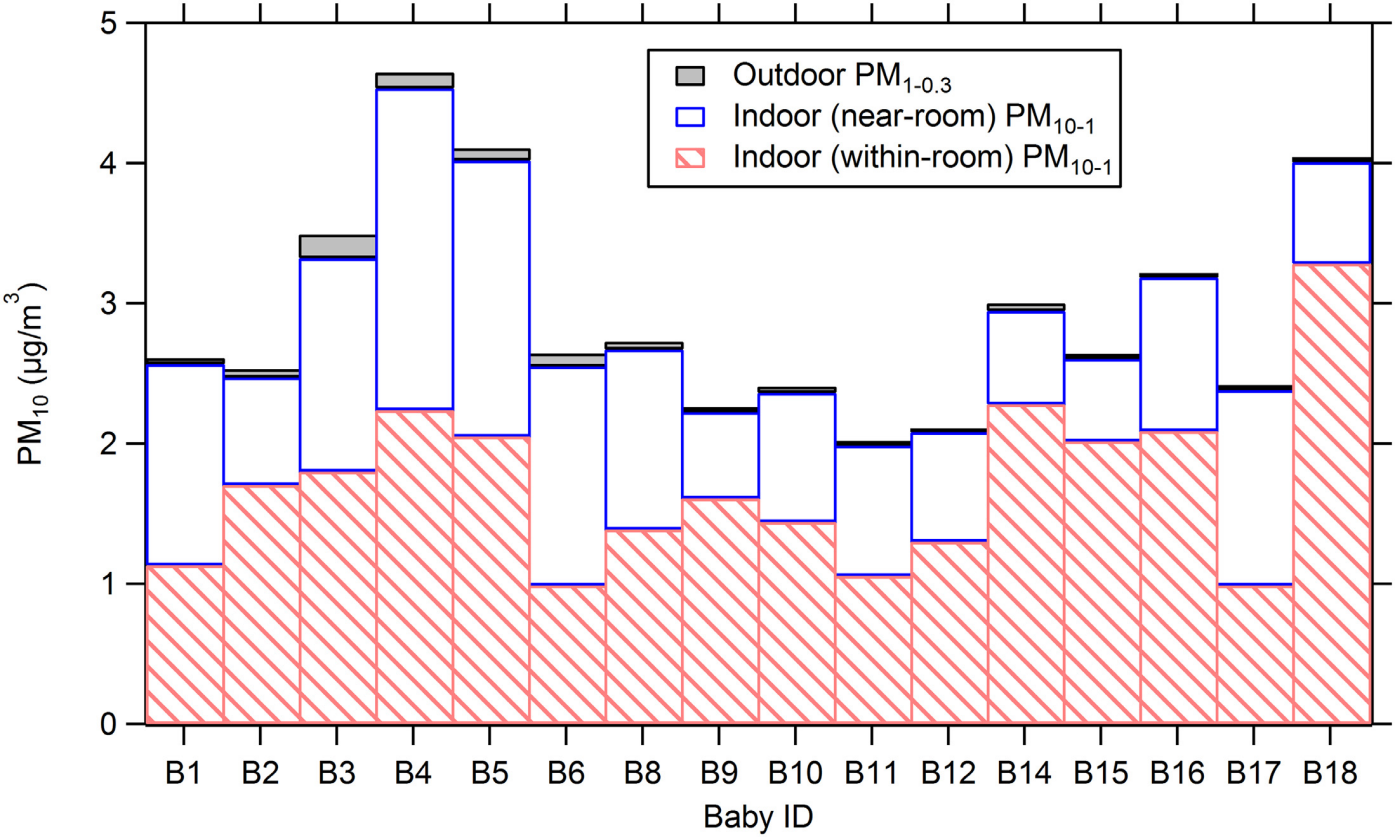
Comparison of individual contributions to PM_10_ mass in the baby rooms from three sources: Indoor (within room) emissions, indoor (near room) emissions, and outdoors.

The indoor particle load consists of a mixture of outdoor particles that have infiltrated indoors (through ventilation supply and via infiltration leaks in the building envelope) and particles directly emitted from indoor sources (from human activities and potentially other processes). Indoor particle concentrations are also governed by filtration, deposition onto indoor surfaces, and removal by means of ventilation [33]. For this study, we divide contributions to indoor particle concentrations in the baby room into three source categories: (1) within-room emissions from occupancy-associated activities; (2) near-room emissions from occupancy-associated activities outside the baby room; (3) and supply from outdoor air. The total particle mass concentrations in the baby rooms were apportioned into these three source categories based on the following inferences: (1) within-room emissions are represented by the average of supermicron particle mass concentrations during the occupied periods minus the supermicron particle mass estimated to have penetrated into the baby room from the near-room spaces; (2); near-room emissions constitute all the supermicron particles in an unoccupied baby room and (3) the source of indoor submicron particles is exclusively outdoor air (an inference that is supported by data in Figs 3 and 5). Fig 6 compares relative source contributions to PM_10_ mass in each baby room. Both within-room and near-room activities contributed strongly to PM_10_ mass in the baby rooms, relative to outdoor air. The influence of within-room activities on elevated PM_10_ mass was more prominent than near-room emissions in most of the baby rooms. Contribution of within-room sources ranged from 37% to 81% across all baby rooms, whereas near-room emissions contributed 18–59% of the total mass concentration. A relatively high near-room particle contribution supports the previous findings [9] that individual neonates who are physically isolated from the human activities occurring outside the room, can be exposed to lower levels of airborne particulate matter. Outdoor air contributed negligibly to PM_10_ mass in the baby rooms, varying in the range 1% to 5%. However, conversely, outdoor air strongly contributed to TPN concentrations, averaging from 82% to 92% across individual baby rooms (S5 Fig).

Coarse particles dominate the PM_10_ mass concentrations in the NICU. Ample evidence from prior literature shows that occupancy-associated emissions through shedding and resuspension cause higher increments of coarse particle loads as compared to fine particles. Larger particles are more likely than smaller particles to become detached from surfaces [34]. Empirically, the rate of indoor emissions is known to increase with particle size when occupants perform more vigorous activities such as walking, cleaning or handling fabrics [35–37].

As illustrated in Fig 6, differences in the contribution of within-room generation of PM_10_ mass were found among the baby rooms. The overall mean of 5-min PM_10_ mass concentrations across all baby rooms was 2.95 μg/m^3^ with a 0.26 coefficient of variation. Possible causes of the variability include differences in the number of occupants and in the activities undertaken in the baby rooms. The occupancy sensors that we utilized could only detect the presence or absence of occupants without providing information about the number of occupants or about the type and intensity of activity performed. For a joint assessment of time-varying occupancy and occupant activity, multiple methods would be needed, i.e. combining metabolic CO_2_ detection, non-directional doorway beam-break sensors and visual observations [38,39]. In a sensitive setting such as the neonatal intensive care unit, the goal of acquiring high quality environmental data must always be tempered with the primary need to not disrupt health-care activities.

## Conclusions

In this year-long study, we investigated the concentrations and sources of size-resolved airborne particles in a normally functioning neonatal intensive care unit. The indoor particle concentrations in the NICU were substantially lower than have been reported for other common occupied indoor environments, such as residences and schools. The contribution of outdoor particles to indoor particle mass concentrations was particularly low owing to the effectiveness of the ventilation system including HEPA filtration. It appears that effective building hygiene protocols also contributed to low indoor airborne particle levels. Although the mass concentration of particles from outdoor air was small, the contribution of ventilation to small particles as assessed by total particle number concentration (considering particles of diameter larger than 0.3 μm) was highly sensitive to the influence of outdoor air, particularly during a period of construction outdoors, which highlights the importance of maintaining efficient particle filtration and limiting air infiltration through the building envelope.

This work has also demonstrated a strong temporal and spatial association between the indoor particle mass concentration (PM_10_) and human occupancy, both considering the temporal pattern in the hospital overall and focusing on infant rooms in particular. The detected particle peaks tied to occupancy were substantially more discernible among larger particles, as would be expected for shedding and resuspension. Conversely, room occupancy contributed little to submicron particle generation. Carbon dioxide levels also exhibited a high spatial correlation across the NICU, but with only a mild dependence on local occupancy.

Within-room emissions made the highest relative source contribution to baby room coarse particle concentrations. Near-room emissions also contributed substantially to baby room coarse particle loads, especially in relation to the small contribution from outdoor air, indicating a possibility to reduce infants’ exposure by further isolating the air in their room from nearby air outside the room. That the occupancy-associated emissions within the room are dominant contributors to airborne particulate concentrations in NICU environments suggests that they may also be an environmental factor influencing infant health. Further evidence supporting this view emerged from our pilot study, revealing that the particle concentrations inside an infant incubator were a substantial proportion of those in room air.

Emerging evidence supports a view that occupancy is an important source of indoor airborne bacteria and fungi. This point has been demonstrated effectively for university [40] and elementary-school classrooms [41]. A recent study in a health-care setting shows a clear connection between indoor air and human-associated bacterial communities [22]. One may reasonably assume that human occupants can be a direct or indirect source of airborne pathogenic microorganisms. It seems worthwhile to consider whether improved nosocomial infection control can be achieved by further limiting bioaerosol emissions associated with occupancy.

This study has identified the prevailing sources and characterized the concentrations of airborne particles in NICU rooms during normal use, but has not considered the mechanisms by which they are transmitted from the room environment to infants. Our study also does not reveal the predominant transmission mechanisms for coarse particle release from occupants in the NICU—whether through resuspension, direct shedding (from skin, hair, clothes, or shoes) or as a transport vector (i.e., what humans bring in from near-room and outdoors). Further research on the emission and transport mechanisms would be important as a basis for developing improved building hygiene protocols. Large spatial and temporal variability in airborne particle levels in the NICU provides evidence of the potential for improving facility management and control practices to further promote the health and welfare of premature infants.

## Supporting Information

S1 Fig Particle number and CO_2_ data acquisition success rate in the hallway and across all baby rooms. The data yield also includes recordings from the nurses’ station and during the HVAC system maintenance that were not included in Table 1.
(TIF)

S2 Fig Comparison of the real-time 1-h mean concentrations of size-resolved particles in the (a) baby room and the nurses’ station; and (b) baby room and the hallway. Time series are shown for 10 sampling days that correspond to B3. The solid line designates particle levels in the baby room, while the shaded area illustrates concentrations at the nurses’ station and in the hallway. Sampling changed from the nurses’ station to the hallway on the date and time demarcated by a vertical dashed line.
(TIF)

S3 Fig Time series (at 5-min resolution) of temperature, relative humidity, TPN and PM10 in a baby room, showing influence of the HVAC filtration system maintenance. These results are based on an analysis of data from 11 February, when filter change-out procedure began at 6:00 AM and lasted for 2.9 hours, as delimited by vertical dashed lines.
(TIF)

S4 Fig Coefficient of determination for the linear correlation between concentrations in two simultaneously sampled baby rooms (B10 and B11) for a nine-day period. The results include 5-min means of small (0.3–0.5 μm) and large (>10 μm) particle number concentrations, and CO_2_ levels.
(TIF)

S5 Fig Comparison of individual contributions to the total particle number concentration (TPN) in the baby rooms from three sources: Indoor (within-room) emissions, indoor (near-room) emissions, and outdoors. Individual source contributions to the total TPN were calculated in the same fashion as explained in relation to Fig 6.
(TIF)

S1 File Nurses’ station and HVAC filter maintenance. (DOCX)

S1 Table Adjustment factors obtained from optical particle counter (OPC) side-by-side tests with a reference instrument OPC3.^1^ (DOCX)

S2 Table Size-resolved particle number concentrations measured in the baby rooms and hallway. The results include data from nurses’ station (for B1, B2 and the first week of B3) and HVAC filter maintenance (no post-processing).
(DOCX)
