# Supplementary figures and images for: Concentrations and Sources of Airborne Particles in a Neonatal Intensive Care Unit
Source: PLoS One. 2016 May 13;11(5):e0154991. doi: 10.1371/journal.pone.0154991 (PMC4866781; doi:10.1371/journal.pone.0154991)

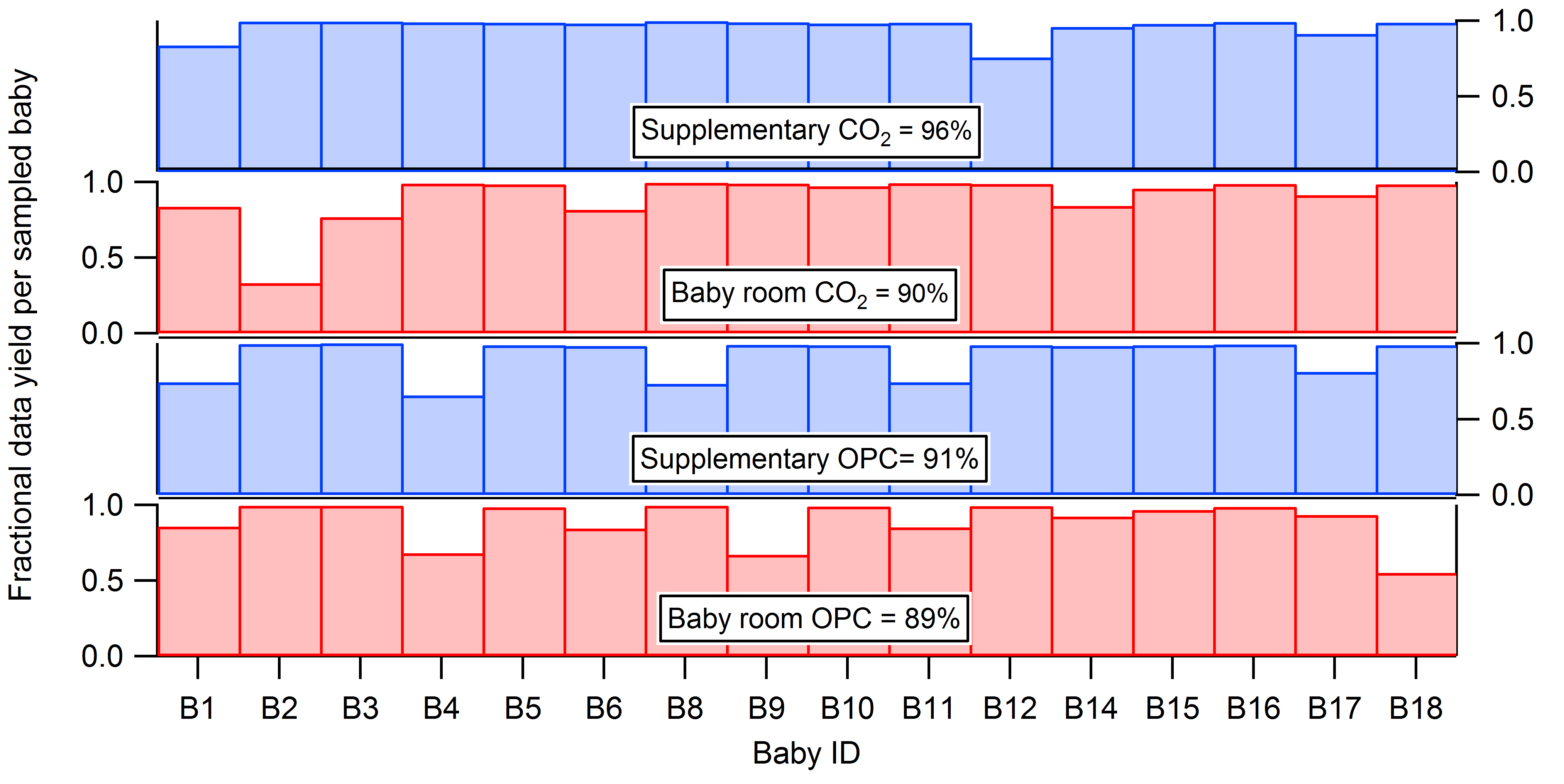

Supplement: S1 Fig — The data yield also includes recordings from the nurses’ station and during the HVAC system maintenance that were not included in Table 1. (TIF) [file pone.0154991.s001.tif]

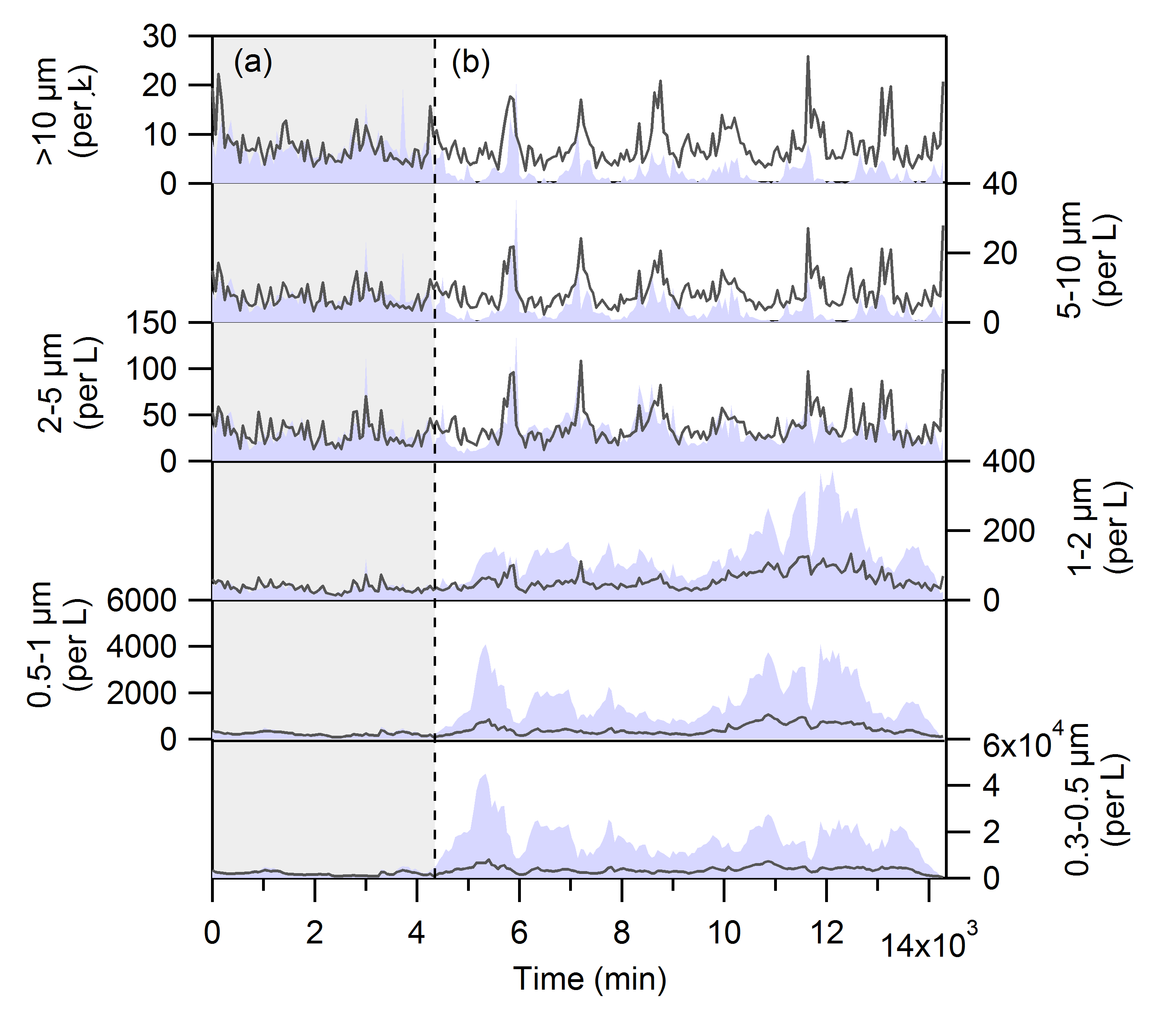

Supplement: S2 Fig — Time series are shown for 10 sampling days that correspond to B3. The solid line designates particle levels in the baby room, while the shaded area illustrates concentrations at the nurses’ station and in the hallway. Sampling changed from the nurses’ station to the hallway on the date and time demarcated by a vertical dashed line. (TIF) [file pone.0154991.s002.tif]

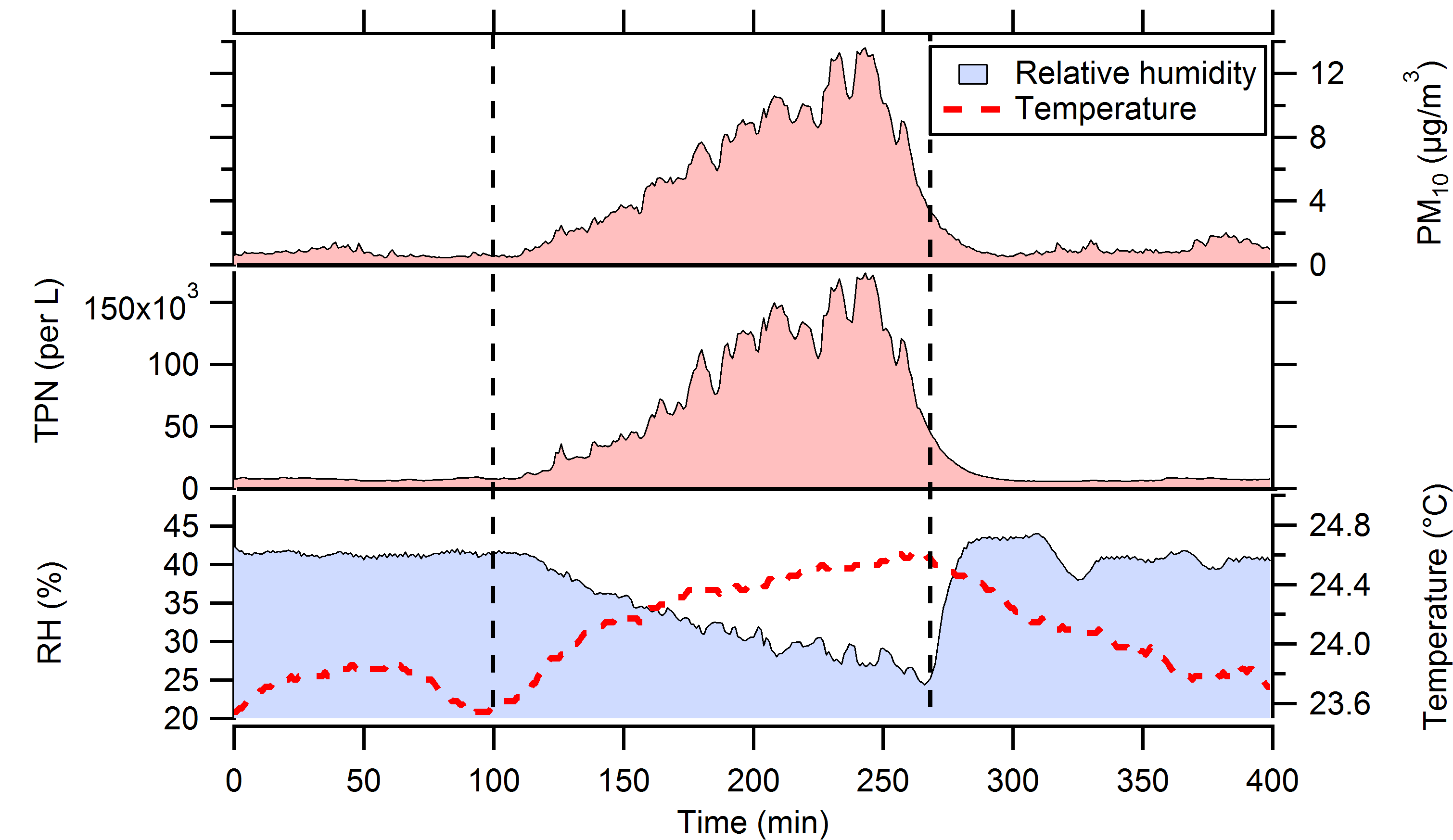

Supplement: S3 Fig — These results are based on an analysis of data from 11 February, when filter change-out procedure began at 6:00 AM and lasted for 2.9 hours, as delimited by vertical dashed lines. (TIF) [file pone.0154991.s003.tif]

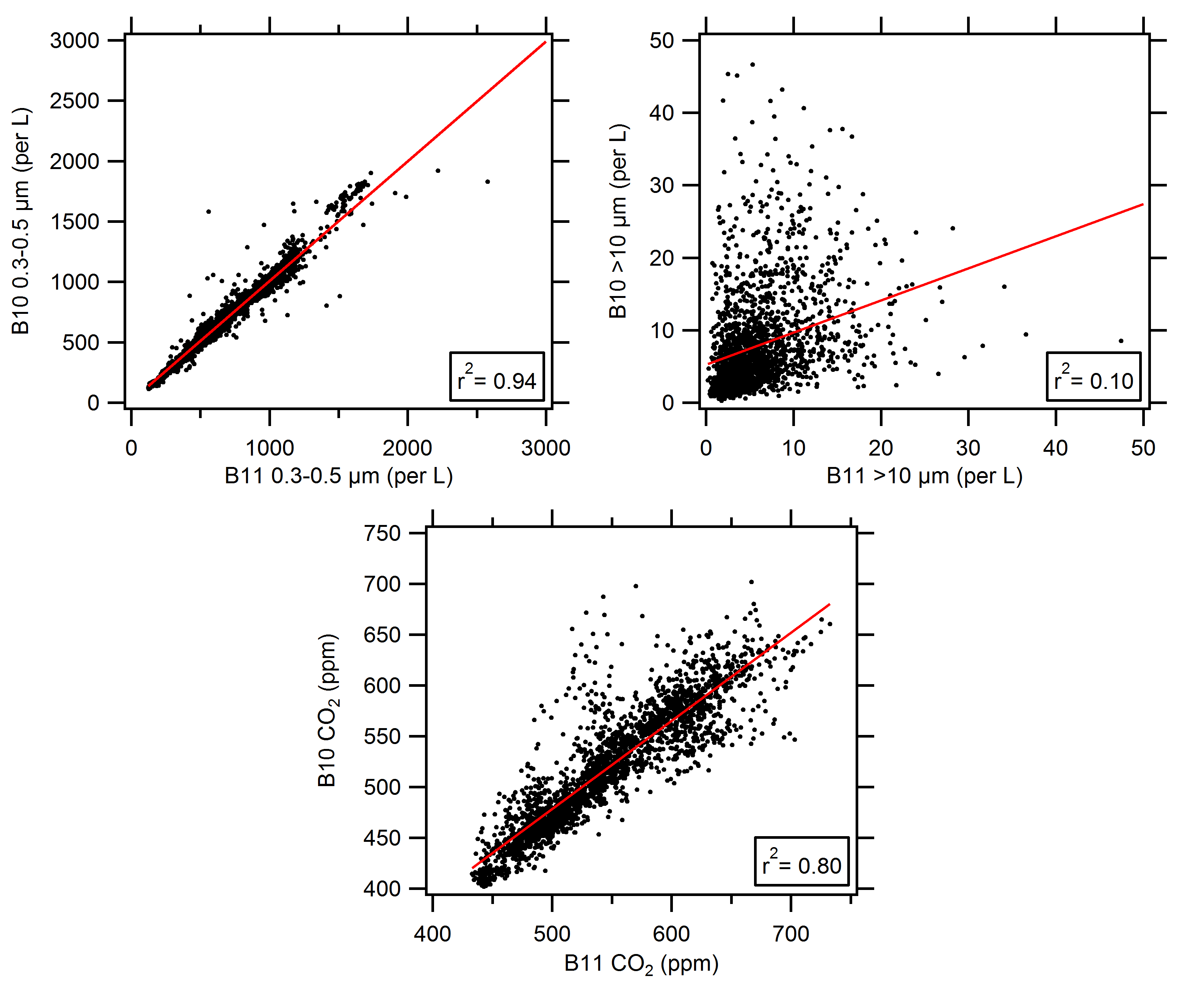

Supplement: S4 Fig — The results include 5-min means of small (0.3–0.5 μm) and large (>10 μm) particle number concentrations, and CO2 levels. (TIF) [file pone.0154991.s004.tif]

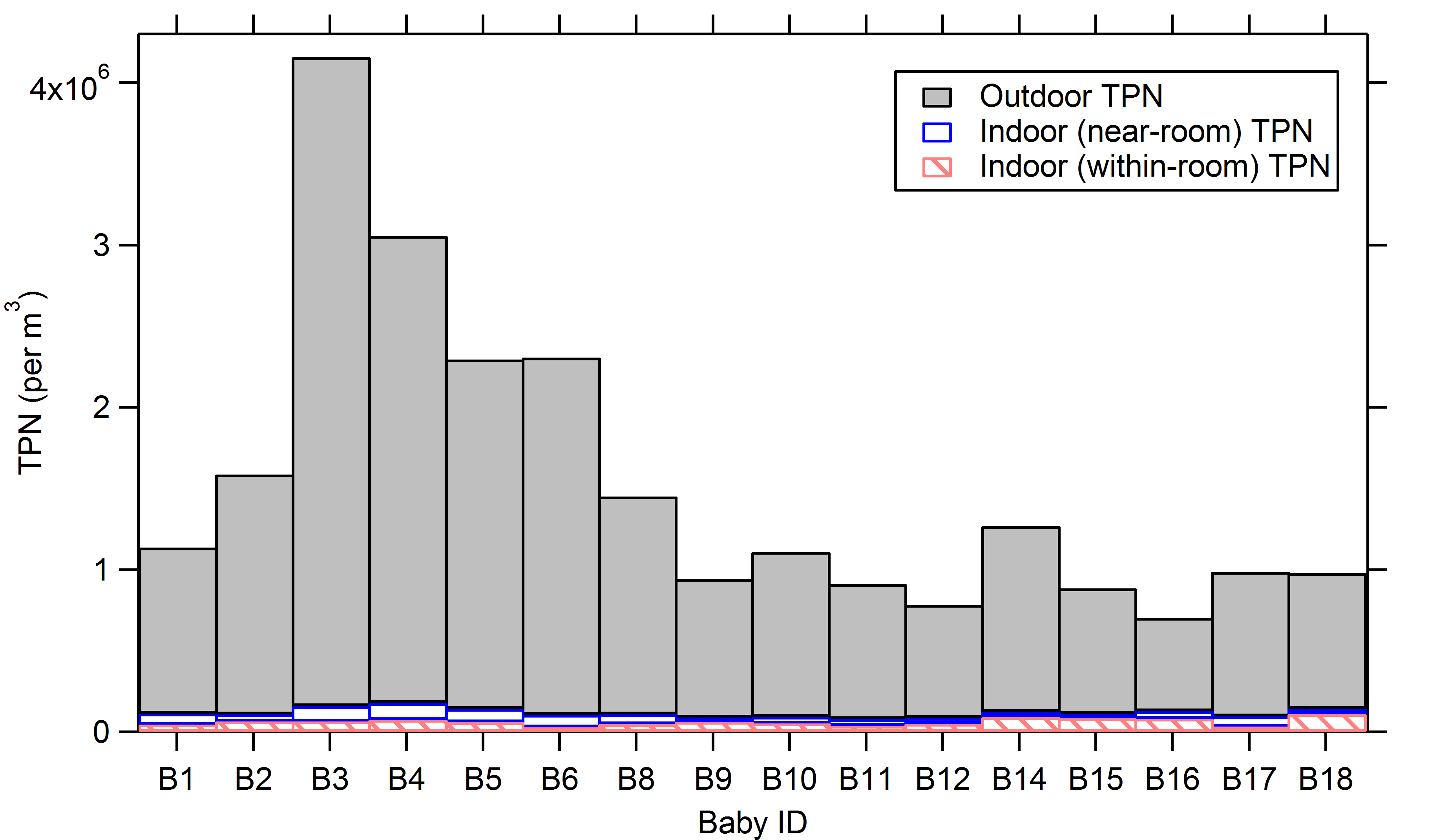

Supplement: S5 Fig — Individual source contributions to the total TPN were calculated in the same fashion as explained in relation to Fig 6. (TIF) [file pone.0154991.s005.tif]
