# Supplementary material for: Concentrations and Sources of Airborne Particles in a Neonatal Intensive Care Unit
Source: PLoS One. 2016 May 13;11(5):e0154991. doi: 10.1371/journal.pone.0154991 (PMC4866781; doi:10.1371/journal.pone.0154991)
Supplement: S1 File — (DOCX) [file pone.0154991.s006.docx]

**S1 File. Nurses’ station and HVAC filter maintenance**

Monitoring in baby rooms B1, B2 and for the first three days of B3 was conducted along with the measurements at the nurses’ station. Relative to baby rooms, the nurses’ station had a higher occupant density, but lower occupant activity, as the hospital personnel were predominantly seated at the nurses’ station. These circumstances are reflected in approximately equal particle number concentrations across the coarse size range in the baby room and nurses’ station (S2 Fig), which highlights the relative importance of both occupant presence and activities performed. The particle levels for the smaller size fractions were also similar, as the supply air was a predominant particle intrusion mechanism. Relocating the sampling instruments from the nurses’ station to the hallway resulted in a sharp increase in the number concentrations of particles smaller than 2 µm, which we presume to be due to the penetration of particle-laden air into the hallway from the stairwell, especially during the period of construction work. Conversely, the number concentration of coarse particles was observed to be lower in the hallway as compared to the nurses’ station, consistent with lower occupant density (S2 Fig).


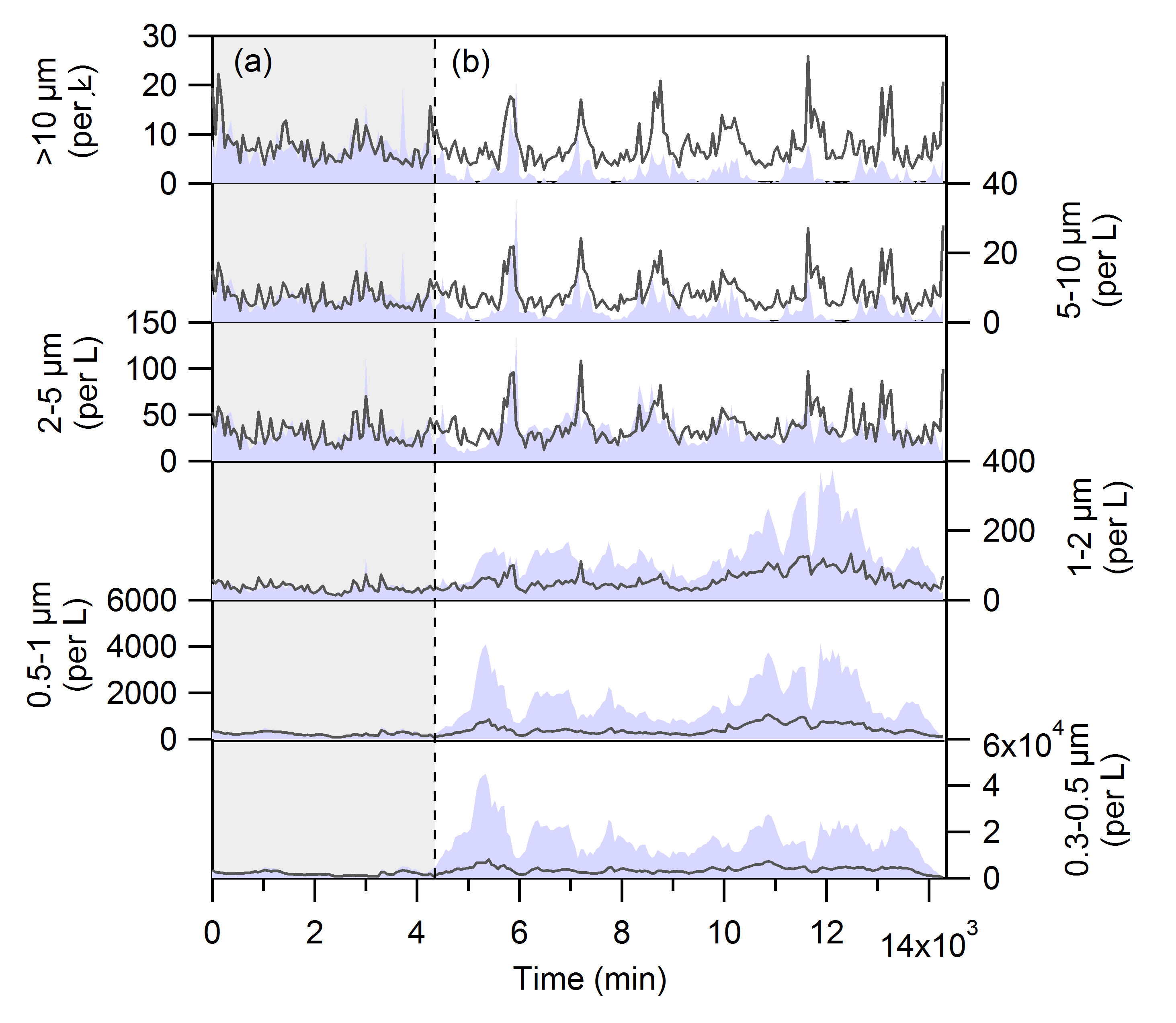


**S2 Fig. Comparison of the real-time 1-h mean concentrations of size-resolved particles in the (a) baby room and the nurses’ station; and (b) baby room and the hallway.** Time series are shown for 10 sampling days that correspond to B3. The solid line designates particle levels in the baby room, while the shaded area illustrates concentrations at the nurses’ station and in the hallway. Sampling changed from the nurses’ station to the hallway on the date and time demarcated by a vertical dashed line.

Maintenance of the HVAC filtration system took place on two separate occasions during the study. S3 Fig. presents time series of temperature, relative humidity, TPN and PM_10_ mass in the baby room during one of the change-out periods for the HVAC filters. Even when the filters were not in place, it appears that the fans continued to run, which is evident from the drop in relative humidity and the spike in particle number concentrations. The PM_10_ mass also exhibited high concentration episodes linked to HVAC filter change out, although the effect was not as pronounced as for TPN. These data indicate that outdoor air contributes not only to TPN, but can also impact coarse particle concentrations in the absence of an effective HVAC filtration system.


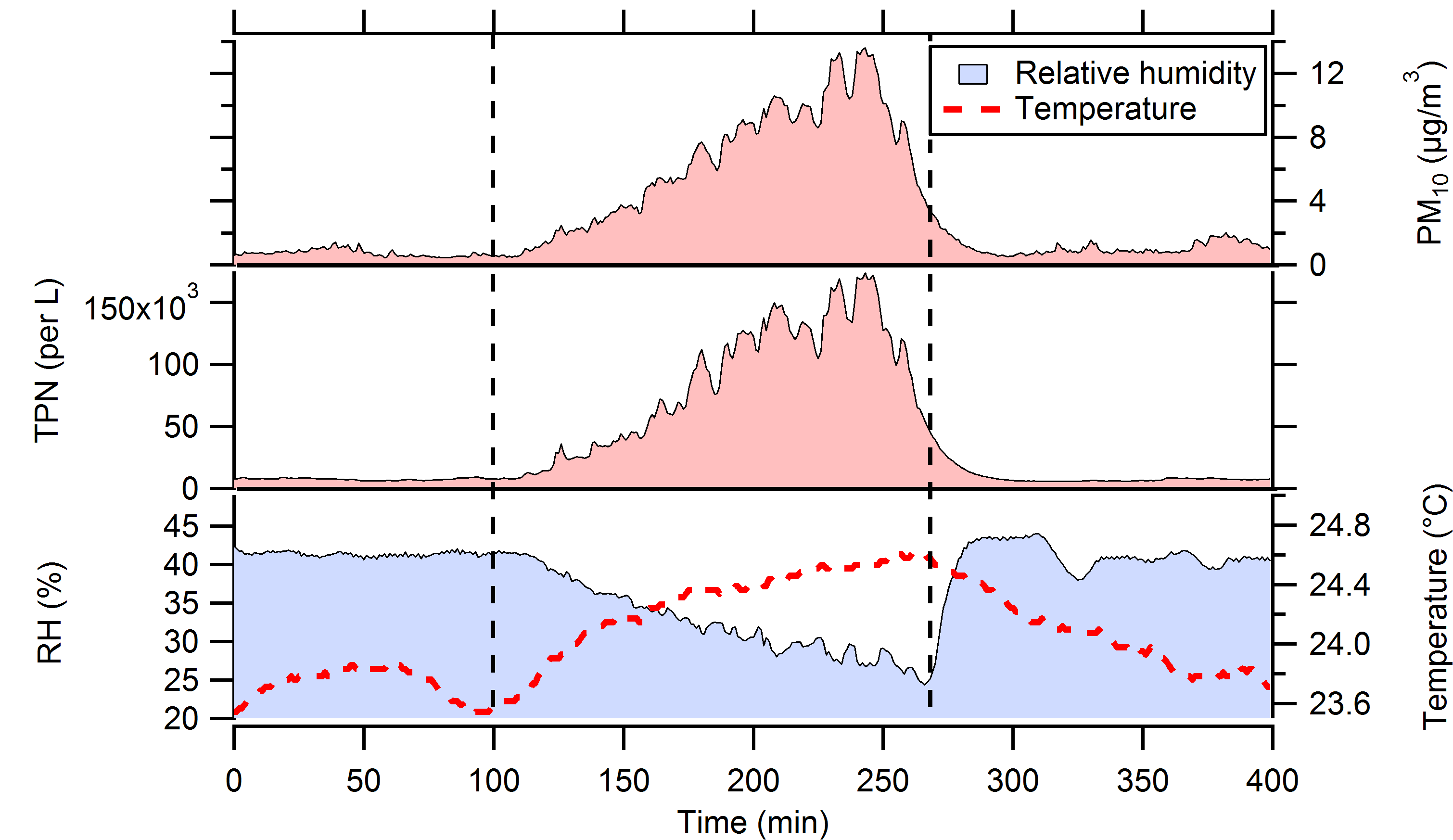


**S3 Fig. Time series (at 5-min resolution) of temperature, relative humidity, TPN and PM10 in a baby room, showing influence of the HVAC filtration system maintenance.** These results are based on an analysis of data from 11 February, when filter change-out procedure began at 6:00 AM and lasted for 2.9 hours, as delimited by vertical dashed lines.
