# Supplementary material for: Concentrations and Sources of Airborne Particles in a Neonatal Intensive Care Unit
Source: PLoS One. 2016 May 13;11(5):e0154991. doi: 10.1371/journal.pone.0154991 (PMC4866781; doi:10.1371/journal.pone.0154991)
Supplement: S1 Table — (DOCX) [file pone.0154991.s007.docx]

**S1 Table.** Adjustment factors obtained from optical particle counter (OPC) side-by-side tests with a reference instrument OPC3.^1^

|  | **Baby 1-5** | | | **Baby 6-9** | | | **Baby 10-14** | | **Baby 15-18** | |
| --- | --- | --- | --- | --- | --- | --- | --- | --- | --- | --- |
| ***d*_p_ size bin (µm)** | **OPC_1_** | **OPC_2_** | **OPC_4_** | **OPC_1_** | **OPC_2_** | **OPC_4_** | **OPC_2_** | **OPC_4_** | **OPC_2_** | **OPC_4_** |
| 0.3 – 0.5 | 0.94 | 0.97 | 0.90 | 0.95 | 0.93 | 0.94 | 0.93 | 0.98 | 0.94 | 0.96 |
| 0.5 – 1 | 0.92 | 0.84 | 0.82 | 0.98 | 0.87 | 0.92 | 0.89 | 0.98 | 0.89 | 0.93 |
| 1 – 2 | 0.69 | 0.77 | 0.82 | 0.73 | 0.80 | 0.84 | 0.74 | 0.84 | 0.69 | 0.83 |
| 2 – 5 | 0.83 | 0.90 | 1.09 | 0.88 | 0.90 | 1.06 | 0.85 | 1.00 | 0.85 | 0.95 |
| 5 – 10 | 0.75 | 0.89 | 0.97 | 0.80 | 0.87 | 0.94 | 0.83 | 0.91 | 0.80 | 0.88 |
| >10 | 0.59 | 0.82 | 1.09 | 0.71 | 0.85 | 0.98 | 0.84 | 0.90 | 0.83 | 0.90 |

^1^Data were compared based on a minute-averaged particle number concentration. The slopes are based on a single parameter linear regression (intercept = 0). OPC_1_ instrument was not employed during the sampling period Baby10-18, hence the adjustments factors are not shown.
