# Supplementary material for: Concentrations and Sources of Airborne Particles in a Neonatal Intensive Care Unit
Source: PLoS One. 2016 May 13;11(5):e0154991. doi: 10.1371/journal.pone.0154991 (PMC4866781; doi:10.1371/journal.pone.0154991)
Supplement: S2 Table — The results include data from nurses’ station (for B1, B2 and the first week of B3) and HVAC filter maintenance (no post-processing). (DOCX) [file pone.0154991.s008.docx]

**Table S2.** Size-resolved particle number concentrations (particles per liter) measured in the baby rooms (BR) and hallway (Hall). The results include data from nurses’ station (for B1, B2 and the first week of B3) and HVAC filter maintenance (no post-processing).

| **Baby**  **ID** | **0.3-0.5 µm** | | **0.5-1 µm** | | **1-2 µm** | | **2-5 µm** | | **5-10 µm** | | **>10 µm** | |
| --- | --- | --- | --- | --- | --- | --- | --- | --- | --- | --- | --- | --- |
|  | **BR** | **Hall** | **BR** | **Hall** | **BR** | **Hall** | **BR** | **Hall** | **BR** | **Hall** | **BR** | **Hall** |
| B1 | 870 | 740 | 97 | 96 | 33 | 34 | 35 | 36 | 7.4 | 8 | 7.5 | 8.2 |
| B2 | 1450 | 1100 | 130 | 170 | 31 | 48 | 28 | 37 | 5.9 | 7.2 | 5 | 6.2 |
| B3 | 3650 | 16700 | 420 | 1650 | 60 | 130 | 38 | 35 | 8.7 | 4 | 7.7 | 2.4 |
| B4 | 2250 | 10600 | 230 | 1150 | 54 | 140 | 56 | 51 | 13 | 5 | 8.2 | 2.1 |
| B5 | 2000 | 14900 | 220 | 1350 | 42 | 120 | 45 | 40 | 13 | 4.4 | 11 | 2.3 |
| B6 | 2150 | 5200 | 250 | 600 | 42 | 64 | 36 | 23 | 8.1 | 3.5 | 5.8 | 2.3 |
| B8 | 1300 | 2200 | 140 | 180 | 32 | 18 | 32 | 11 | 7.8 | 2.7 | 5.9 | 2.1 |
| B9 | 690 | 830 | 62 | 61 | 17 | 9 | 18 | 7.5 | 4.3 | 1.9 | 4.1 | 1.6 |
| B10 | 880 | 1000 | 95 | 95 | 25 | 14 | 27 | 11 | 6.5 | 2.6 | 7.9 | 2.4 |
| B11 | 700 | 810 | 73 | 71 | 21 | 12 | 24 | 11 | 5.6 | 2.8 | 5.9 | 2.6 |
| B12 | 610 | 830 | 63 | 59 | 21 | 10 | 23 | 10 | 5.3 | 2.4 | 5.2 | 2.3 |
| B14 | 850 | 1150 | 99 | 130 | 22 | 12 | 22 | 8.1 | 5.1 | 1.9 | 4.9 | 2 |
| B15 | 650 | 860 | 76 | 67 | 27 | 10 | 26 | 7.3 | 6.5 | 1.9 | 5.9 | 1.8 |
| B16 | 450 | 680 | 61 | 57 | 24 | 11 | 28 | 8.6 | 6.6 | 2.3 | 5.8 | 2.2 |
| B17 | 780 | 1000 | 90 | 82 | 29 | 12 | 32 | 8.2 | 7.1 | 2 | 6.6 | 1.8 |
| B18 | 790 | 1150 | 84 | 95 | 22 | 11 | 27 | 7.8 | 6.8 | 1.8 | 5.7 | 1.6 |
| **Mean** | **1250** | **3750** | **140** | **370** | **31** | **41** | **31** | **20** | **7.4** | **3.4** | **6.4** | **2.7** |
| **S.D.** | **570** | **2150** | **60** | **220** | **10** | **20** | **8** | **7** | **2** | **1** | **1** | **1** |
